# Assessing reservoir heterogeneity using the turbulence factor as an effective tool for hydraulic flow unit classification for BM-85 Well, Gulf of Suez, Egypt

**DOI:** 10.1038/s41598-026-37379-0

**Published:** 2026-02-18

**Authors:** Ibrahim M. Al-Alfy, Marwa Z. El-Sawy, Nouran S. Salama, Osama M. Elnaggar

**Affiliations:** 1 Exploration Division, Nuclear Materials Authority, Cairo, Egypt; 2 Exploration Department, Egyptian Petroleum Research Institute (EPRI), Nasr City, Cairo, Egypt; 3 Geology Department, Faculty of Science, Capital University, Cairo, Egypt

**Keywords:** Reservoir heterogeneity, Turbulence factor, Hydraulic flow unit and pore throat radii, Energy science and technology, Engineering, Hydrology, Solid Earth sciences

## Abstract

Reservoir heterogeneity is crucial for predicting fluid flow behavior and improving hydrocarbon recovery in mature and complex reservoirs. This study investigates the heterogeneity of the Lower Senonian Formation in the BM-85 well, Gulf of Suez, Egypt, with a focus on classifying hydraulic flow units (HFUs) using the turbulence factor (β) as a key diagnostic parameter. Core data and well logs were used to predict some of pore throat parameters and porosity, respectively, which were integrated with turbulence factor calculations. Well-log interpretation, supported by litho-saturation crossplots, successfully delineated two pay zones. Pay 1 is characterized by moderate porosity (21%) and hydrocarbon saturation (63%), while Pay 2 exhibits excellent reservoir quality with an average permeability of 716.3 mD, effective porosity of 18–21%, and hydrocarbon saturation of 44%. Reservoir vertical and lateral heterogeneity is characterized using continuous, high-resolution downhole measurements. The wide permeability range, from 0.1 to 6431.82 mD, reflects strong reservoir heterogeneity and flow capacity. The Dykstra–Parsons coefficient (V = 0.91) confirmed extreme heterogeneity, indicative of pronounced contrasts in permeability and pore-throat distribution. The RQI–β cross-plot reveals the presence of two distinct hydraulic flow units within the studied reservoir interval, reflecting strong heterogeneity. High-quality flow units are associated with low β values and high permeability, whereas tight intervals show elevated β values and restricted flow behavior. The turbulence factor (β), calculated using the most recent empirical formulation, effectively captures non-Darcy flow effects and highlights subtle pore-system variations that are not resolved by conventional porosity–permeability relationships alone. These results demonstrate that incorporating β into HFU classification provides a more physically meaningful and robust framework for reservoir characterization, with direct implications for optimizing pay-zone identification and development strategies in the Gulf of Suez and other highly heterogeneous carbonate/clastic reservoirs.

## Introduction

Rudeis Formation is a significant oil-producing reservoir in the Gulf of Suez. This formation exhibits significant lateral facies variations, making it a challenging exploration target due to tectonic complexity. The studied region predominantly consists of shale, interspersed with occasional limestone layers, but primarily comprises fine-grained marl and sandstone^1^.

Reservoir heterogeneity refers to the non-uniformity within a reservoir, both laterally and vertically, that arises from multiple geological factors^2^. It reflects variations in rock properties such as porosity, permeability, and fluid saturation caused by differences in depositional environments, subsequent diagenetic alterations (e.g., compaction, cementation, dissolution), and structural modifications introduced by later tectonic movements. These combined influences lead to a complex spatial distribution and turbulence architecture, making reservoir prediction, characterization, and exploitation more challenging. Research on reservoir heterogeneity has progressed from conventional reservoirs (e.g., carbonates and deltas) to unconventional ones (e.g., tight sandstone/shal), focusing increasingly on its influence on residual oil distribution and overall reservoir performance^3–5^.

Reservoir heterogeneity in the Gulf of Suez is strongly influenced by its unique tectono-stratigraphic evolution, which is closely tied to rifting and fault block formation associated with the Red Sea–Suez rift system. The reservoirs are dominated by Miocene clastics and pre-rift carbonates, where lateral and vertical facies changes, syn-rift faulting, and differential subsidence create significant variations in thickness and reservoir quality^6^. Diagenetic processes, including cementation, dissolution, and dolomitization, further enhance the heterogeneity, resulting in complex porosity–permeability distributions. Fault-bounded compartments and tilted blocks often act as both conduits and barriers to fluid flow, which complicates hydrocarbon migration and recovery strategies. Consequently, the Gulf of Suez is considered one of the most structurally complex and heterogeneous petroleum provinces in Egypt, requiring detailed reservoir characterization for efficient field development and enhanced oil recovery^7,8^.

Hydraulic flow unit (HFU) characterization using turbulence-based parameters, such as flow zone indicators and permeability–porosity relationships, has been widely applied to improve reservoir quality assessment and flow prediction. However, most previous turbulence-based HFU studies emphasize empirical petrophysical clustering with limited consideration of the geological and diagenetic controls governing pore-system evolution. As a result, HFU boundaries are often defined statistically rather than being anchored to pore-scale processes, which limits their predictive capability, particularly in heterogeneous carbonate reservoirs. This study provides one of the first detailed HFU characterizations specifically focused on tight to moderately permeable reservoir intervals in the Gulf of Suez. By quantifying how turbulence-related parameters vary across different HFUs and tying these variations to rock fabric and diagenetic modification, the study offers new insight into flow heterogeneity and connectivity in reservoirs that are commonly considered marginal or unconventional.

The fluid distribution, flow characteristics, and reservoir recovery effect are all directly impacted by reservoir heterogeneity, according to domestic researchers, which is the foundation of the fluid properties and production characteristics inside the rock space.

Reservoir heterogeneity, as a fundamental attribute reflecting the storage and flow capacity of reservoirs, has become a central focus of oil and gas research both domestically and internationally. Its significance lies in its ability to characterize variations in seepage behavior within reservoirs, which directly impacts production performance. Understanding reservoir heterogeneity is crucial for resolving contradictions encountered during oilfield development, particularly those related to uneven displacement and low recovery efficiency^9^. In addition, heterogeneity exerts a strong influence on the effectiveness of water flooding operations, the spatial distribution of remaining oil, and the overall hydrocarbon storage potential. Consequently, it is considered a key geological factor in optimizing reservoir management strategies, improving recovery technologies, and ensuring more accurate prediction of reservoir performance^10^.

This paper provides a comprehensive review of reservoir heterogeneity by examining its research progress, classification systems, and quantitative characterization methods as reported in a wide range of literature. Particular attention is given to the diverse methodologies applied to quantitatively analyze reservoir heterogeneity using petrophysical, and field outcrop core data, which together enable a more accurate depiction of reservoir architecture and fluid flow behavior by classification the Reservoirs using turbulence flow that helps refine the understanding of flow mechanisms, especially in complex or tight reservoirs by incorporating turbulence effects using models like Forchheimer’s equation and the β-factor.

Traditional methods like Dykstra–Parsons quantify heterogeneity; they provide limited insight into the dynamic flow behavior within the pore network. In contrast, the turbulence factor (β) serves as a dynamic parameter that directly links pore structure to fluid flow, capturing non-Darcy effects and subtle variations not resolved by conventional porosity–permeability relationships. This provides a novel and physically meaningful tool for hydraulic flow unit (HFU) classification.”

**Fig. 1:**
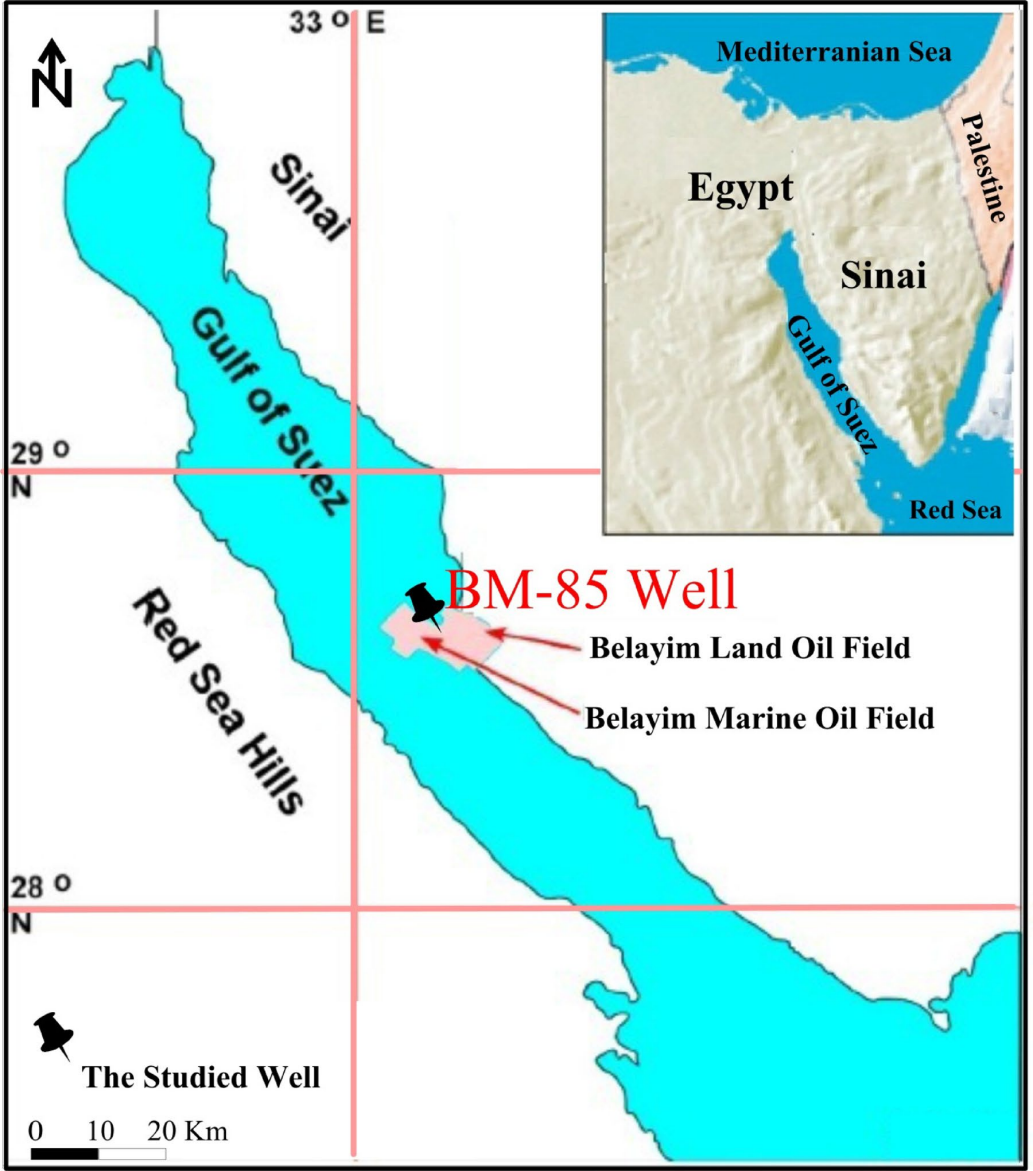
Location map of the studied well, Gulf of Suez, Egypt.

In this investigation, a total of 103 core plug samples obtained from the Lower Senonian Formation in the BM-85 well, Gulf of Suez, Egypt (Fig. 1), were systematically examined to describe and assess the reservoir characteristics. Complementary to the core analyses, a suite of well-logging data comprising gamma ray, density, sonic, resistivity, and neutron measurements was integrated to provide a comprehensive evaluation of the reservoir. The log data were further interpreted through litho-saturation crossplots, which enabled the vertical assessment of lithological variations, porosity, and fluid saturation trends within the studied interval.

The Gulf of Suez, where reservoirs are characterized by strong heterogeneity and complex structural settings, this approach enhances the prediction of fluid flow behavior, supports more accurate simulation models, and ultimately contributes to more efficient field development and improved hydrocarbon recovery.

## Aim of study

The aim of this study is to assess reservoir heterogeneity within the Lower Senonian Formation of the BM-85 well, Gulf of Suez, Egypt, utilizing the turbulence factor as an effective tool for hydraulic flow unit (HFU) classification. The study intends to demonstrate how turbulence factor analysis can improve the understanding of pore system dynamics, refine the delineation of flow units, and provide a more accurate prediction of reservoir performance compared to conventional porosity–permeability approaches.

### Highlights

- Integration of core and well log data to characterize reservoir heterogeneity in the BM-85 well, Gulf of Suez.
- Application of the turbulence factor as a diagnostic tool for hydraulic flow unit (HFU) classification.
- Identification of distinct HFUs reflecting variations in flow reservoir heterogeneity.
- Turbulence factor shown to outperform conventional porosity–permeability relationships in detecting subtle pore system variations and explain the effect of flow and storage capacity.
- Investigated the impact of heterogeneity on reservoir quality, providing improved insight into fluid flow dynamics in the Lower Senonian Formation.

## Methodology

Reservoir classification using turbulence flow is a confirming tool used to evaluate and classify reservoirs based on the dynamic behavior of fluid movement through the porous media, particularly focusing on non-Darcy flow regimes. The flow behavior is better captured using the Forchheimer equation, which incorporates both viscous (linear) and inertial (quadratic) terms to account for departures from Darcy’s law in high-velocity regimes^11^. Furthermore, studies have successfully utilized a turbulence factor (β) to differentiate flow units and pore systems in reservoir rocks revealing that samples with comparable porosity can exhibit markedly different flow characteristics owing to variations in pore-network structure and diagenetic history^12^. The following is a brief discussion of the methods used in this study.

### Core data analysis

Routine core analyses combined with well-logging techniques are widely regarded as essential methods for evaluating the petrophysical characteristics of reservoir rocks. Routine core analysis (RCAL) provides fundamental measurements of porosity and permeability which are critical for determining reservoir quality and assessing heterogeneity. When integrated with log data, these analyses enhance the accuracy of reservoir characterization and classification^13–15^. For the petrophysical studies using core analysis data, a total of 103 plug samples were selected representatively for description of formation by helium porosity (∅He) and permeability in two orthogonal directions (vertical kV and horizontal kH) measurements. In order to determine whether or not production would be profitable given the reservoir’s capacity to store and distribute hydrocarbons, it is basically necessary to define and estimate the reservoir quality^16^. The reservoir quality index (RQI, in µm) that has been proposed and tested by many authors was applied for evaluating the Formation’s samples as recommended by many authors^17–19^. Prior to interpretation, the logs were calibrated and validated against the available conventional core analysis data.

### Well logging data integrated

The current methodology focused on evaluating the Lower Senonian Formation in BM-85 well (Gulf of Suez, Egypt) using a whole set of conventional well logs available for this interval. These included gamma ray, density, neutron, sonic, and resistivity measurements, which together provide an integrated framework for reservoir evaluation to compute key petrophysical parameters. The gamma ray log was employed to distinguish between shale and sand lithologies, while the density and neutron logs were cross-plotted to estimate porosity and identify potential gas effects. Sonic log data were utilized to derive acoustic transit times and confirm porosity estimations, whereas resistivity logs were interpreted to determine hydrocarbon saturation and fluid distribution^20,21^. Through the integration of these datasets, the petrophysical parameters of the Lower Senonian Formation were systematically computed, offering insights into its lithological variability, reservoir quality, and heterogeneity.

Beyond to estimating hydrocarbon and water saturations, the petrophysical evaluation incorporated the determination of shale volume as well as both total and effective porosity. The interpretation of these parameters was carried out using the Techlog 2015.2 software program, which allowed systematic data processing and cross-plot analysis. Water saturation (Sw) was quantified by applying the Indonesian equation developed by^22^, which accounts for the influence of shale on resistivity responses in complex lithologies. Porosity (∅e) and shale volume (Vsh) were computed through neutron–density log tools and gamma ray readings respectively, providing reliable estimates of lithological variations and reservoir quality. This integrated workflow enhances the accuracy of reservoir characterization by simultaneously considering lithology, porosity, fluid content, and shale distribution within the studied interval.

### Reservoir heterogeneity quantification

Reservoir heterogeneity, a key factor controlling fluid flow and recovery efficiency, was quantitatively assessed using the Dykstra–Parsons coefficient (V), a statistical approach developed by^23^ to characterize the spread of permeability values within a given reservoir zone. The V-value is calculated from the cumulative frequency distribution of permeability measurements and serves as an indicator of the degree of heterogeneity present in the formation. This method involves plotting the permeability values in descending order on a logarithmic probability scale to establish a permeability distribution curve. From this curve, K₅₀ (the permeability at 50% cumulative probability) and K₈₄.₁ (the permeability at 84.1% cumulative probability) are determined. The coefficient V is then calculated using the formula:

$$\:\mathrm{V}=(\mathrm{K}50-\mathrm{K}84.1)/\mathrm{K}50$$

where: k_50_ is the median permeability, while k_84.1_ is the percentile of a set of permeability data arranged in decreasing order.

Following the classification scheme proposed by^19,24^, the V values were interpreted as follows: an extremely heterogeneous reservoir (1.0 ≥ V > 0.75), a highly heterogeneous reservoir (0.75 ≥ V > 0.50), a moderately heterogeneous reservoir (0.50 ≥ V > 0.25), a slightly heterogeneous reservoir (0.25 ≥ V > 0.10), and a homogeneous reservoir (0.10 ≥ V). This classification provides insight into the degree of permeability contrast across different reservoir intervals. Higher V-values indicate a greater spread in permeability values, often reflecting complex depositional environments, diagenetic alterations, or facies variability. In contrast, lower V-values suggest more uniform permeability, typical of well-sorted, homogeneous sandstone.

### Calculation of key parameters

The RQI is a dimensionless parameter that correlates porosity and permeability to characterize a rock’s ability to transmit fluids. It is calculated using the equation:

$$\:\:RQI=0.0314\surd\:\frac{\boldsymbol{K}}{\boldsymbol{\varPhi\:}}$$

where the reservoir quality index (RQI) is measured in microns. The normalized cumulative reservoir quality index can be computed and plotted against depth to divide the reservoir into flow units^25^.

Non-Darcy flow is one of the most significant causes of reducing the productivity of hydraulically fractured high rate wells, according to^21,26^. This phenomena has been extensively investigated by other authors^27,28^ using field case situations.

The turbulence factor, often denoted as β, is a parameter used in reservoir rock characterization to quantify the deviation from Darcy’s Law, particularly in cases of high flow rates or when pore structure is complex. It’s used to characterize the flow system heterogeneity in the reservoir rocks by accounting for non-Darcy flow behavior caused by the rock’s turbulence^29^. At higher flow rates, the linear relationship between pressure drop and flow rate described by Darcy’s Law breaks down. This non-linear behavior is often observed in reservoirs with complex pore structures or high flow rates. This behavior is strongly influenced by the pore structure of the rock; factors like pore size distribution, pore throat geometry, and the presence of fractures all affect the magnitude of the inertial forces and thus the value of β. While they aren’t a direct measure of heterogeneity, the turbulence factor can provide insights into the impact of heterogeneity on fluid flow^30^.

In essence, the turbulence factor (β) is a useful tool for understanding and quantifying the impact of pore structure and heterogeneity on fluid flow in reservoir rocks, especially when non-Darcy effects are significant. Reservoir heterogeneity, with its variations in permeability and pore structure, can significantly affect fluid flow behavior. The turbulence factor helps quantify how these heterogeneities influence the non-Darcy flow regime. The turbulence factor (β) is often used alongside other parameters like permeability, porosity, and various heterogeneity indices to provide a comprehensive description of reservoir characteristics. A higher value of β generally indicates a more complex pore structure and potentially higher heterogeneity^31^.

Numerous writers have examined and computed the turbulence factor, including^32–35^. In 1950^36^, introduced a single traditional parameter to characterize the distribution of permeability within a reservoir pay zone. Using a graphical representation (Lorenz plot), they defined the Lorenz coefficient of heterogeneity as:

$${\text{Lorenz coefficient}}={\mathrm{Area}}\;{\mathrm{(ABCA)}}/{\mathrm{Area}}\;({\mathrm{ADCA}})$$

This coefficient ranges from zero, representing a reservoir with perfectly uniform permeability, to values approaching unity in highly heterogeneous systems.

Numerous empirical correlations have been proposed in the literature for estimating the turbulence (non-Darcy) factor (β). The turbulence (non-Darcy) flow coefficient (β) was recalculated using an updated empirical relationship that reflects recent experimental and core-scale investigations of inertial flow effects in porous media where this formulation is consistent with current turbulence-based HFU methodologies and was applied uniformly across all samples to ensure comparative consistency rather than site-specific calibration. The turbulence factor (β) used in this study was estimated using the empirical relationship according to^35^:

$$\:{\upbeta\:}=a{k}^{-b}=\:{10}^{10.264}{k}^{-0.85}$$

Where β is the turbulence factor, *k* is permeability (mD), and *a* and *b* are empirically derived constants. Based on recent studies, the exponent *b* typically ranges between 0.5 and 1.0, reflecting the strong inverse dependence of inertial resistance on permeability and pore-throat size in low- to moderate-permeability reservoir rocks.

The Reservoir Quality Index (RQI) was plotted against the turbulence factor (β) to facilitate the classification of reservoir rock types based on both flow capacity and pore structure characteristics^12^. This cross-plot allows for the integration of porosity and permeability data (through RQI) with the textural complexity and flow behavior (represented by β), providing a more robust framework for distinguishing between different reservoir quality classes.

Classification of a reservoir based on pore throat radius is a key method used to evaluate reservoir quality, fluid flow capacity, and production potential. Pore throat radius controls how easily fluids can move through the pore network, especially in tight or heterogeneous formations. This classification is often linked to reservoir rock types, capillary pressure behavior, and flow units. Estimated pore throat sizes (R^35^) from core-based porosity-permeability properties have been done^37^.

A widely used approach is the Reservoir Quality Index (RQI) or the Winland r^35^ method, which uses Mercury Injection Capillary Pressure (MICP) data to derive the pore throat radius. The Winland r^35^ value (pore throat radius at 35% mercury saturation) is a standard metric that is classified by^38–40^. The following list of five rock types is closely related to reservoir potential: Types 1–2: r^35^, which are usually good conventional reservoirs, range between 1 and 2.5 and are distinguished by good reservoir quality and good to exceptional flow capacity. Type 3: R^35^ has a value between 0.5 and 1. It can produce at moderate pressure or after stimulation, and its characteristics include rocks of moderate reservoir quality and limited flow capacity. Type 4–5: Unconventional or tight reservoirs (r^35^ > 0.5) that need advanced completion or fracturing. This classification is used in reservoir zonation and flow unit identification, Hydrocarbon recovery forecasting, Cutoff determination for net pay, Understanding water saturation and capillary pressure behavior and Fracture design in tight reservoir^40^.

In our research, some of Mercury Injection Capillary Pressure (MICP) was derived by using imperial equations to quantify the reservoir behavior, stepwise laboratory measurements of porosity and permeability are performed, porosity is measured using techniques such as helium expansion or Boyle’s law porosity, while permeability is assessed using gas or liquid flow methods under steady-state or pulse-decay conditions^21^. This allows for the construction of pressure-dependent porosity–permeability curves, which are crucial for understanding reservoir compaction, stress sensitivity, and production behavior under reservoir or overburden stress conditions. Based on these measurements, the characteristic pore-throat radius at 35% mercury saturation (r^35^) was calculated using the Winland equation, expressed as:

$$\:\mathrm{log}\left({\mathrm{r}}^{35}\right)=0.732+0.588\mathrm{log}\left(k\right)-0.864\mathrm{l}\mathrm{o}\mathrm{g}\left(\varPhi\:\right)$$

Where r^35^​is the pore-throat radius (µm), k is permeability (mD), and ϕ is porosity (fraction). In addition, pore-size distribution characteristics were interpreted using Pittman’s correlations, which relate MICP-derived capillary pressure data to permeability and pore-throat geometry.

Flow capacity and storage capacity are two fundamental measures used to describe reservoir heterogeneity. The modified stratigraphic Lorenz technique^41^ refines the storage–flow capacity relationship by adding depth information. Flow capacity represents the ability of the rock to transmit fluids, it related to permeability × thickness (k*h) of each layer. A layer with high permeability contributes more to flow capacity, even if its pore volume is small. Storage capacity represents the ability of the rock to store fluids. It related to porosity × thickness (φ*h) of each layer. This relationship is quantified using the Lorenz coefficient or Dykstra–Parsons coefficient. A thick bed that has low permeability may store a lot of fluid however not much of it can move. High-permeability streaks contribute disproportionately more to flow capacity compared to their share of storage capacity. The greater the separation, the higher the heterogeneity. This makes it possible to differentiate Hydraulic Flow Units (HFUs), assess their vertical contributions, and improve reservoir characterization and flow predictions^42^.

## Results and discussion

**Fig. 2:**
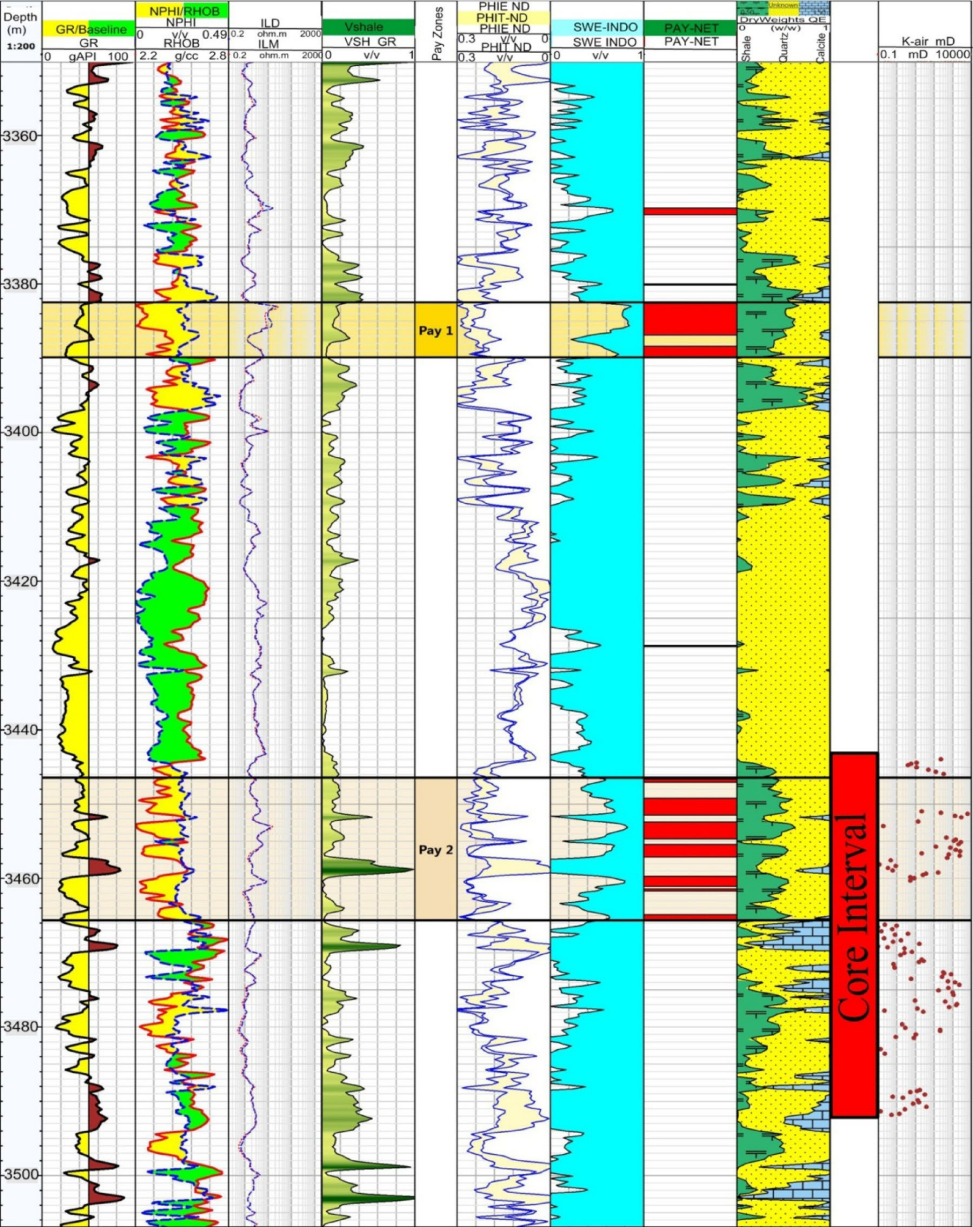
Litho-saturation cross plot showing the input data and the results of the studied sequences.

Well logging is a critical tool in evaluating reservoir heterogeneity, which refers to the variability in reservoir properties (such as porosity, permeability, and lithology) both vertically and laterally. This variability significantly affects fluid flow, reservoir quality, and hydrocarbon recovery. The petrophysical evaluation of the reservoir sequences includes determining effective and total porosity, shale volume, and both water and hydrocarbon saturations have been done and showed in vertical litho-saturation crossplot (Fig. 2). The calibrated litho-saturation plot illustrates variations in lithology, as well as water and hydrocarbon content derived from the calculated shale volume, effective porosity, and water saturation for the studied Formation. The range of gamma ray readings in track 2 indicates a light change along the reservoir; the low value is roughly 10.8, and the greatest value does not surpass 88 api. These values reflect a slightly variation in lithology, the majority along the reservoir is sandstone. To calculate the average of the reservoir parameters needed for the petrophysical analysis, cutoff values for shale volume, porosity, and water saturation were set at 35%, 10%, and 50%, respectively. These reservoir parameters indicated discrimination the sequences into two pay zones. The two pay zones were selected based on integrated cutoff criteria, including low shale volume, sufficient effective porosity, low water saturation, positive net pay indication and favorable lithofacies and permeability trends. The upper reservoir interval (Pay 1), extending from 3382.47 to 3389.82 m, exhibits an average hydrocarbon saturation of 63%, an effective porosity of 21%, and a shale volume of about 15% as showed in Table 1 with facies track indicates dominance of clean sandstone with limited shale interbeds. The lower interval (Pay 2), with a net pay thickness of 19.34 m, is predominantly oil-bearing and is defined by an average hydrocarbon saturation of 44%, a very low shale content of 0.19%, and an effective porosity of 18%. The rock facies track shows stacked reservoir facies with localized shale streaks, explaining minor porosity–permeability fluctuations.

**Table 1:** Conventional core data and reservoir quality parameters of the studied BM-85 well, Gulf of Suez, Egypt.

	Φ, %	Kh, mD	RQI, µm	r35, µm	β, m⁻¹	SW, F	VSH, F	PHIE, F
Group A								
Min	10.89	0.1	0.027	0.14	3.06E + 10	0.18	0.01	0.01
Max	29.14	50.52	0.43	3.18	6.24E + 15	1.00	0.97	0.32
Avg	20.23	8.64	0.16	1.10	4.54E + 14	0.84	0.28	0.12
Group B								
Min	0.69	126.53	21.22	5.54	4,659,385	0.33	0.01	0.05
Max	4.66	6431.82	32.57	50.72	6.19E + 09	1	0.75	0.26
Avg	1.93	1312.66	26.75	18.76	8.96E + 08	0.73	0.18	0.17

In the second pay zone, the presence of core data within this interval provides direct validation of reservoir quality and confirms the petrophysical interpretation. Permeability measurements indicate predominantly high values, with an average of 716.3 mD. Porosity evaluation shows that fluid-filled porosity obtained from the Dean–Stark method averages 29.9%, while helium porosity averages 21.1%. The close agreement between these core-derived parameters and the well log interpretation confirms the reliability of the petrophysical evaluation and emphasizes the excellent reservoir quality of this interval. This integrated approach ensures that both Pay-1 and Pay-2 represent true productive intervals rather than isolated log anomalies. Although Pay-2 exhibits slightly more heterogeneity than Pay-1, the integrated petrophysical response confirms it as an effective pay zone with viable flow potential.

The investigated core interval between 3444.07 and 3491.88 m comprises a total of 103 samples, representing a significant section of the reservoir. Given the significant variation in permeability values, which range from as low as 0.1 mD to as high as 6431.82 mD, a thorough investigation was conducted to identify and evaluate the geological and petrophysical factors responsible for these discrepancies (Fig. 2). These variations may be attributed to differences in grain size, sorting, cementation, pore structure, and the degree of compaction, as well as the presence of fractures or clay content within the reservoir rock. A wide distribution of porosity is evident, extending from 11 to 33%.

**Fig. 3:**
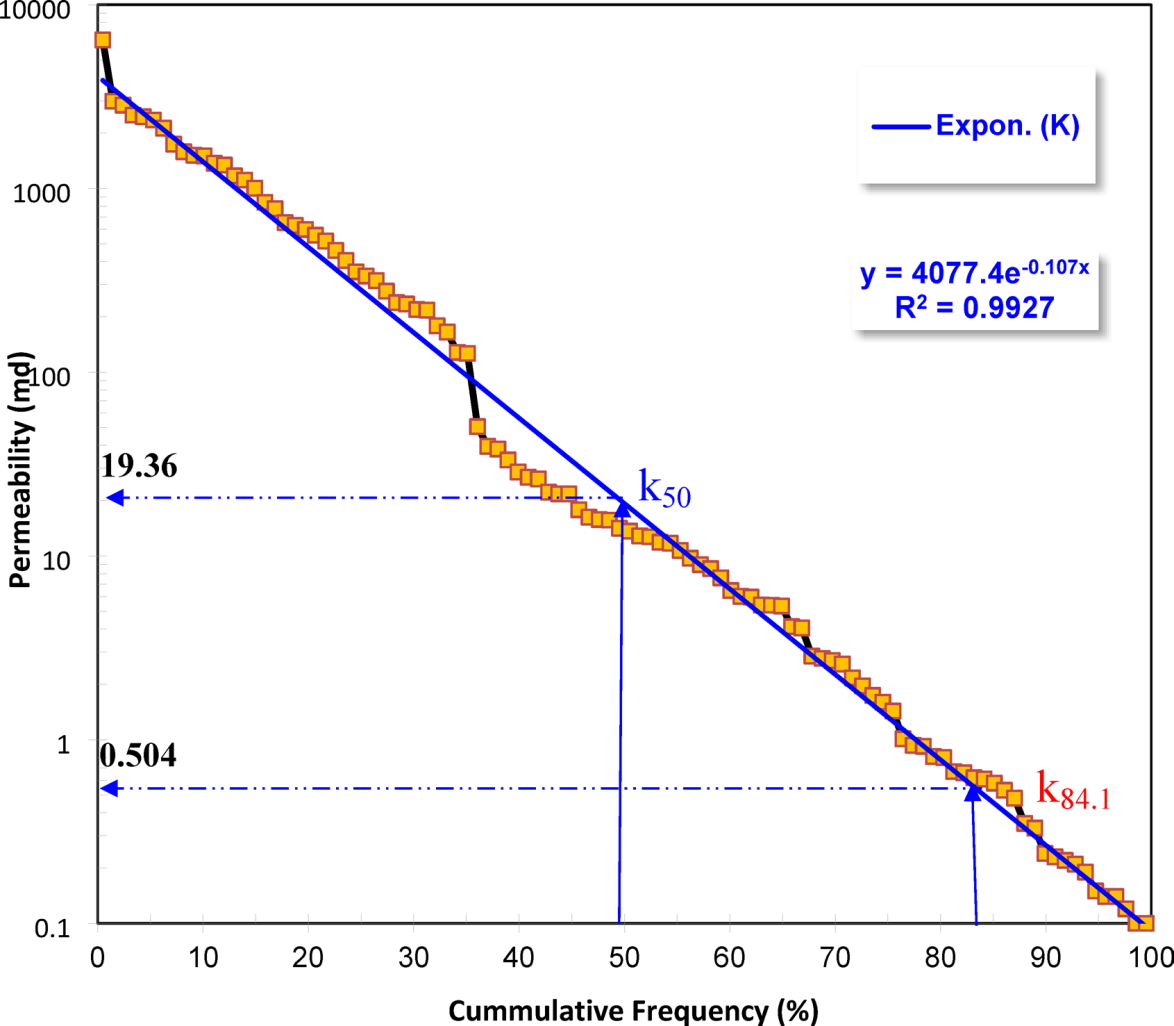
Estimating the heterogeneity degree of the measured permeability using Dykstra-Parsons technique (1950).

According to the^18^ method, the permeability distribution of the studied sequence is characterized by extreme heterogeneity (Table 1), as evidenced by a heterogeneity index of V = 0.91 (Fig. 3). This high value reflects a wide contrast in permeability between different reservoir layers, indicating poor uniformity and strong stratification within the sequence. Also, such a high value suggests significant variation in pore-throat sizes and flow capacity among the reservoir intervals, indicating poor permeability uniformity and uneven fluid distribution during flow.

**Fig. 4:**
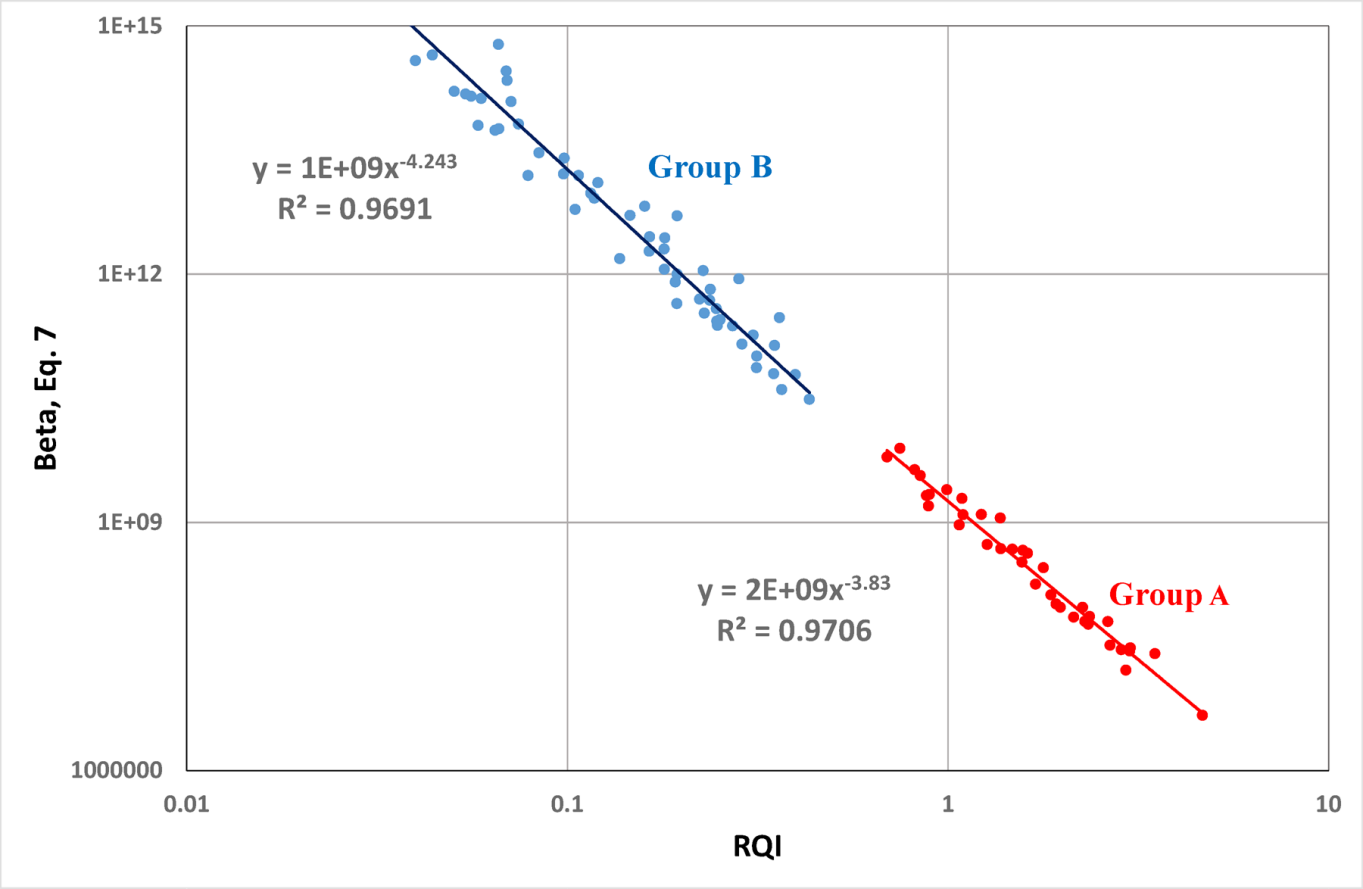
Reservoir Quality Index (RQI) versus turbulence factor (β) plot to classify samples into hydraulic flow units.

The Reservoir Quality Index (RQI) versus turbulence factor (β) cross-plot (Fig. 4) is an advanced petrophysical technique employed to evaluate the degree of reservoir heterogeneity and classify hydraulic flow units (HFUs). Each HFU defines a distinct zone of consistent reservoir behavior, where pore-throat sizes, connectivity, and flow properties are relatively uniform, regardless of variations in lithology or depositional fabric. This classification enables the grouping of intervals with similar hydraulic characteristics, thereby providing a more accurate framework for reservoir characterization. By integrating porosity, permeability, and pore geometry, the RQI–β relationship provides a more nuanced classification of reservoir rock types than porosity or permeability alone. Figure 4 demonstrates a clear distinction between reservoir rock types based on the RQI–β relationship. Rocks with high RQI and low β values (Table 1), such as those in HFU 1 (R² = 0.97), represent high-quality reservoir zones characterized by efficient pore connectivity, good permeability, and enhanced flow capacity. These intervals typically act as the main fluid conduits within the reservoir. In contrast, rocks with low RQI and high β values, represented by HFU 2 (R² = 0.97), correspond to tighter formations with poorly connected pores, limited permeability, and reduced flow efficiency. This contrast between HFU 1 and HFU 2 reflects the heterogeneous nature of the studied sequence, where high-quality reservoir intervals coexist with lower-quality, flow-restrictive zones. Such differentiation is crucial for identifying productive intervals, predicting reservoir performance, and guiding effective development and recovery strategies.

**Fig. 5:**
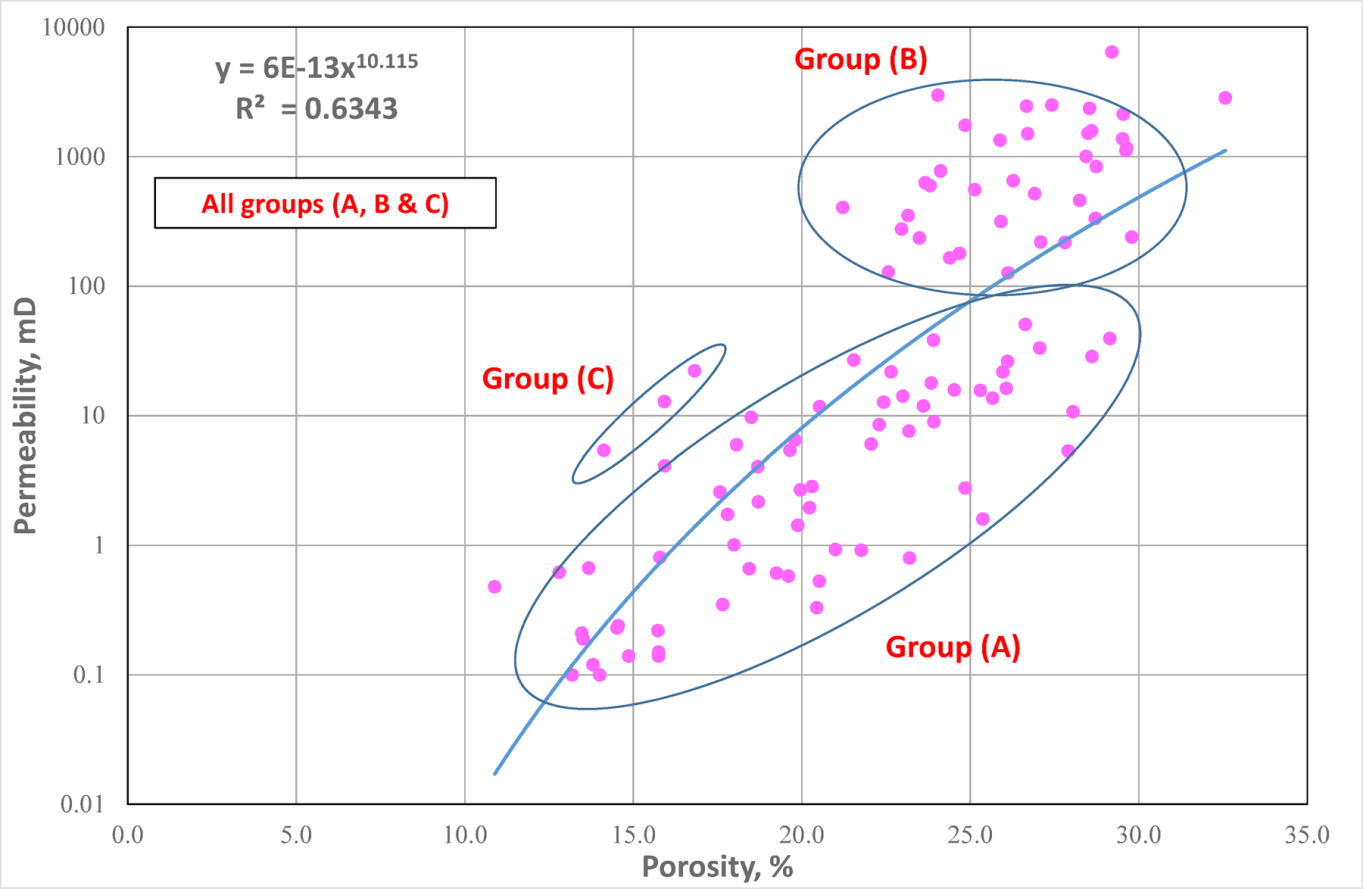
Plotting horizontal permeability as a function of helium porosity confirming samples differentiation into two main groups using turbulence factor (β).

Horizontal permeability (kH) was cross-plotted against effective porosity (∅He) (Fig. 5) to assess the relationship between pore space and fluid flow potential within the reservoir. This cross-plot serves as a diagnostic tool to evaluate how effectively porosity contributes to permeability, a key factor controlling reservoir quality and productivity. The cross-plot reveals an overall positive correlation (R² = 0.6343), which can be divided into three groups, with two main Groups (A and B) representing the dominant groups while third group (group c) is represented by only three data points and does not constitute a statistically meaningful population. As a result, this minor cluster was considered negligible and was not included in the detailed discussion. Using 18% effective porosity as a threshold value, this cross plot clearly and meaningfully separates the investigated core plugs into two primary groups. The samples were divided into two groups because samples with porosity more than 22% typically shown a noticeable variation in permeability.

In Group B, the positive correlation suggests that higher porosity and permeability enhances the rock’s capacity to transmit fluids, reflecting well-connected pore networks with minimal pore-throat restrictions, better reservoir quality, and more effective fluid flow channels. On the other hand, Group A has low permeability but relatively high porosity. Significant scatter or a weaker connection suggests that secondary factors like pore shape, diagenetic processes (such cementation or dissolution), clay mineral concentration, or microfracturing are at effect. These factors can obstruct or restrict pore connectivity, thereby reducing flow efficiency despite high pore volume. This variability underscores the heterogeneous nature of the studied reservoir and highlights the importance of integrating porosity–permeability relationships with other petrophysical tools for accurate reservoir characterization.

**Fig. 6:**
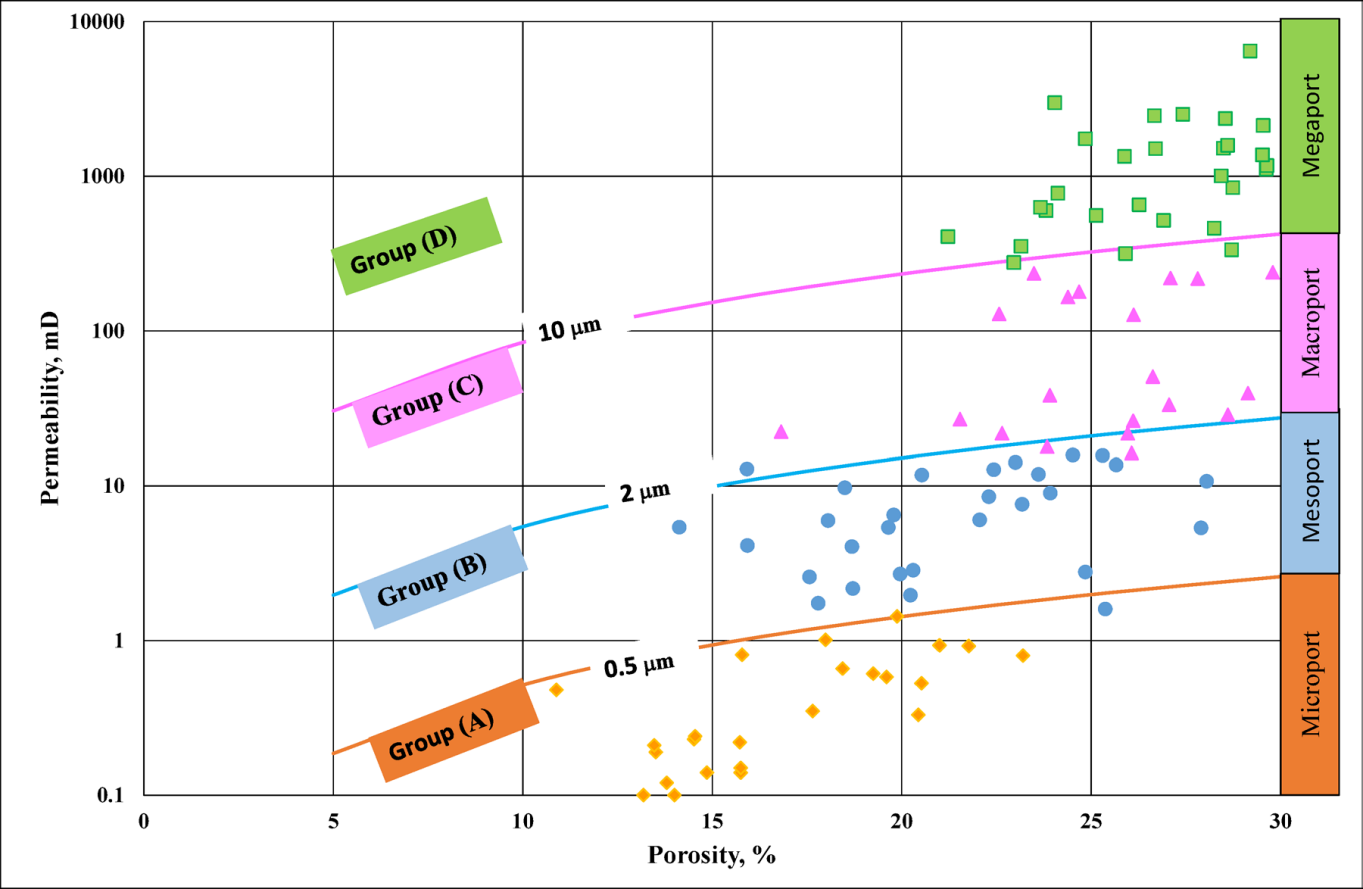
Classification of samples into groups according to pore throat radius.

Based on MICP analysis and pore throat radii (r^35^) (Fig. 6), the reservoir was classified into four quality groups: A (microport), B (mesoport), C (macroport), and D (megaport). Groups C (r^35^ > 2 μm) and D (r^35^ > 10 μm) represent reservoirs with excellent properties, high porosity, high permeability, and strong flow potential (equivalent to group B in Fig. 4). In contrast, groups B (r^35^ < 2 μm) and A (r^35^ < 0.5 μm) correspond to tight formations with low effective permeability and poor producibility without stimulation, (equivalent to group A in Fig. 4) (Table 1). This classification reflects a wide spectrum of reservoir qualities, from extremely tight to highly productive intervals.

**Fig. 7:**
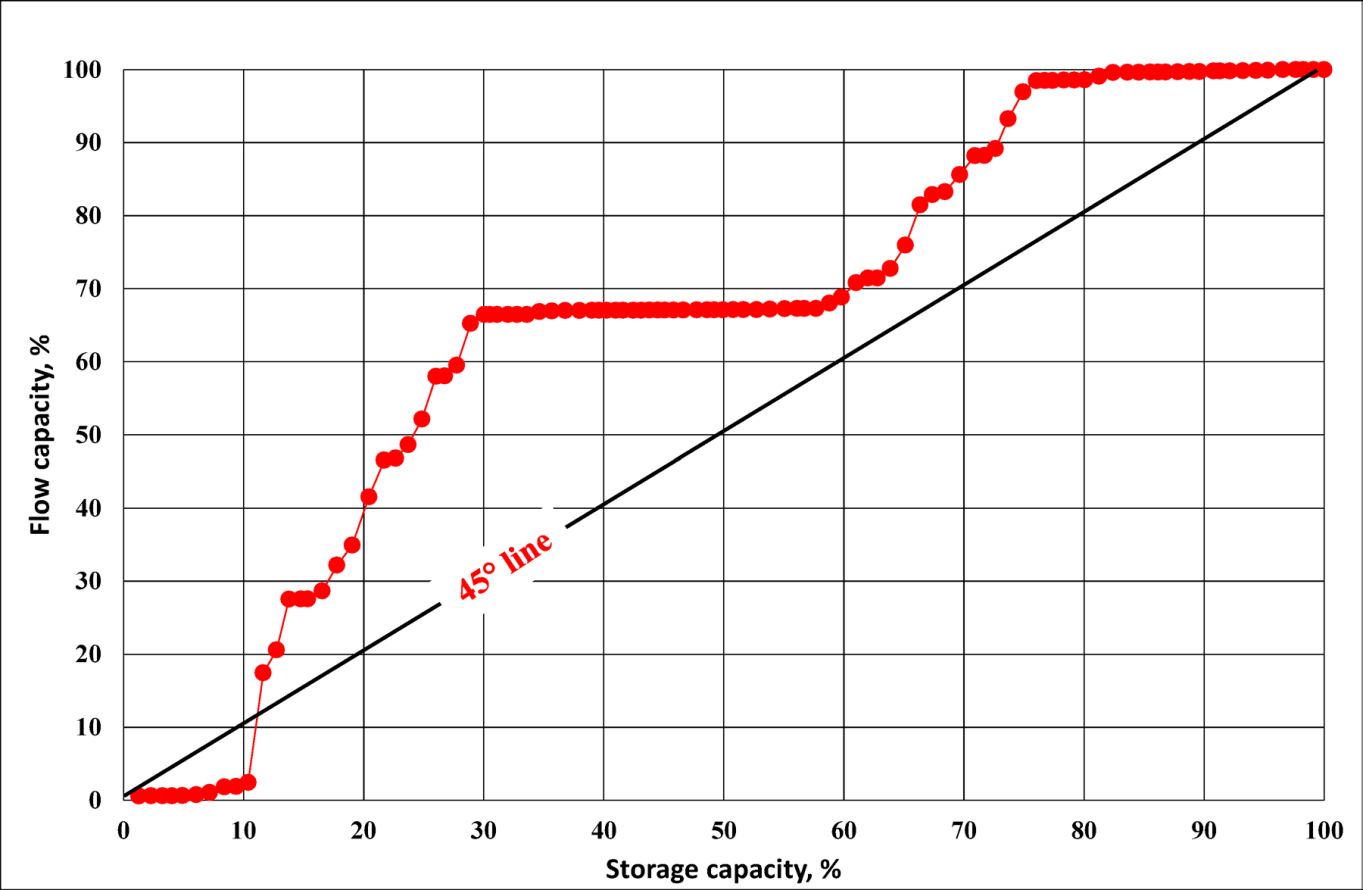
Lorenz plot for displaying permeability heterogeneity of the studied samples.

The stratigraphic Lorenz methodology was presented by^41^ as an alternate method to plot the flow capacity (represented by the permeability) as a function of the storage capacity (represented by the porosity) in order to assist in sorting the investigated samples into various HFUs. It may be possible to estimate the contribution of each flow unit to the overall flow capabilities by taking the depth into consideration (Fig. 7). This figure of flow capacity versus storage capacity shows that the samples being studied were added up into two flow units. A somewhat zigzag line in the stratigraphic modified Lorenz plot, which shows the presence of various intercalations of relatively varying flow capacities through this flow unit, illustrates the heterogeneity of HFU-1 and 2.

## Summary and conclusion

This study provides a detailed investigation into reservoir heterogeneity within the Lower Senonian Formation of the BM-85 well, Gulf of Suez, through the integration of core analyses, well-log interpretation, and advanced heterogeneity characterization techniques. Reservoir heterogeneity, driven by depositional environments, diagenetic alterations, and structural evolution, was shown to exert a fundamental control on porosity–permeability distributions, fluid flow behavior, and overall reservoir performance. Well-log interpretation, supported by litho-saturation crossplots, successfully delineated two pay zones. Pay 1 is characterized by moderate porosity (21%) and hydrocarbon saturation (63%), while Pay 2 exhibits excellent reservoir quality with an average permeability of 716.3 mD, effective porosity of 18–21%, and hydrocarbon saturation of 44%. The strong consistency between core-derived parameters and log-based interpretations highlights the robustness of the integrated workflow.

Petrophysical evaluation from core analysis revealed wide ranges of porosity (11–33%) and permeability (0.1–6431.82 mD), reflecting strong lithological and textural variability. The Dykstra–Parsons coefficient (V = 0.91) confirmed extreme heterogeneity, indicative of pronounced contrasts in permeability and pore-throat distribution. The integration of Reservoir Quality Index (RQI) with the turbulence factor (β) allowed classification of Hydraulic Flow Units (HFUs), successfully distinguishing high-quality reservoir zones with well-connected pore networks from tighter, flow-restrictive intervals. Cross-plots of porosity versus permeability further demonstrated the strong influence of pore structure, diagenesis, and clay content on flow efficiency, emphasizing the role of secondary controls in governing reservoir performance.

Mercury Injection Capillary Pressure (MICP) measurements provided additional insights into pore-throat radii (r^35^) and allowed classification into distinct reservoir quality groups ranging from tight to highly productive. Flow–storage capacity relationships, analyzed using the modified stratigraphic Lorenz technique, confirmed that high-permeability streaks disproportionately contribute to flow capacity, underscoring the complexity of fluid distribution in heterogeneous systems.

Overall, the study demonstrates that reservoir heterogeneity in the Gulf of Suez is controlled by the interplay of depositional, diagenetic, and structural processes. Quantitative tools such as the Dykstra–Parsons coefficient, RQI–β cross-plots, and pore-throat classification provide powerful means of assessing heterogeneity, classifying flow units, and predicting reservoir behavior. These findings highlight the importance of integrating core, log, and laboratory data to improve reservoir characterization and guide optimal field development strategies. Understanding the spatial distribution of heterogeneity is critical for enhancing recovery efficiency, managing waterflooding operations, and accurately forecasting reservoir performance in one of Egypt’s most structurally complex petroleum provinces. Permeability, heterogeneity, and the reservoir quality index have a major effect on the turbulence factor, a useful indicator for reservoir zonation based on flow properties. Additionally, the distribution of pore throat sizes has a positive/negative effect on the flow system; the more macropores there are, the higher the permeability and lower the turbulence factor, and the more micropores there are, the lower the permeability and higher the turbulence factor. Finally, Pay Zone 1 corresponds to HFU-1 (Group A), characterized by moderate porosity and permeability, while Pay Zone 2 aligns with HFU-2 (Group B), exhibiting higher reservoir quality and flow capacity. This correlation highlights the consistency between log-based and core-based analyses, providing a more integrated understanding of reservoir heterogeneity and reinforcing the relevance of HFU classification in delineating productive intervals. We now emphasize that reservoirs dominated by macropores exhibit lower turbulence factor (β) values, reflecting more efficient flow behavior, whereas tighter pore systems are associated with higher β values and increased non-Darcy flow effects.

## Data Availability

Data will be available on reasonable request by contacting the corresponding author: [Marwa\_epri@yahoo.com](mailto: Marwa_epri@yahoo.com) .

## Declarations

### Permission of data

Permission and consent were obtained from the Egyptian General Petroleum Corporation for conducting the field study in the Lower Senonian Formation in the BM-85 well, Gulf of Suez, Egypt.

### Declarations of generative AI and AI-assisted technologies in the manuscript preparation process

During the preparation of this work the author(s) used [chatGPT] in order to [revise the manuscript]. After using this tool/service, the author(s) reviewed and edited the content as needed and take(s) full responsibility for the content of the published article.

## References

[1] Ayyad, H. M., Fathy, M., Hewaidy, A. G. A. & Abdallah, A. Sequence stratigraphy of the burdigalian rudeis formation in Ras El-Ush oil field, Gulf of suez: application of gamma-ray analysis and biostratigraphy. *Mar. Pet. Geol.* **122**, 104694. 10.1016/j.marpetgeo.2020.104694 (2020).

[2] Li Jie, M. et al. Study on heterogeneity of Chang 21 reservoir in Zhengsi block of Ordos basin [J]. *Yunnan Chem. Technol.* **50** (12), 123–127 (2023).

[3] Zhan, H., Fang, F., Li, X., Hu, Z. & Zhang, J. Shale reservoir heterogeneity: A case study of Organic-Rich longmaxi shale in Southern Sichuan. *China Energies*. **15** (3), 913. 10.3390/en15030913 (2022).

[4] Guo, R. A review of methods for studying reservoir heterogeneity. *Acad. J. Sci. Technol.* **14**, 1 (2025).

[5] Xu, H., Hao, Y. & Niu.B., Huand. L., Zhang. L., and Study on the influence mechanisms of reservoir heterogeneity on flow capacity during fracturing flooding development. *Energies* **18** (13), 3279. 10.3390/en18133279 (2025).

[6] Bosworth, W. & McClay, K. R. : Structural and stratigraphic evolution of the Gulf of Suez rift, egypt: a synthesis. In (eds Ziegler, P. A., Cavazza, W., Robertson, A. H. F. & Crasquin-Soleau, S.) Peri-Tethys Memoir 6: Peri-Tethyan Rift/Wrench Basins and Passive Margins (567–606). Mémoires du Muséum National d’Histoire Naturelle, Paris. (2001).

[7] Younes, M. A. & McClay, K. R. Development of accommodation zones in the Gulf of Suez–Red sea Rift, Egypt. *AAPG Bull.* **86** (6), 1003–1026. 10.1306/61EEDB0C-173E-11D7-8645000102C1865D (2002).

[8] Khalid, M. S. et al. Carbonate reservoir characterization and permeability modeling using machine learning a study from Ras Fanar field, Gulf of Suez, Egypt. *Earth Sci. Inf.* **17**, 4675–4694. 10.1007/s12145-024-01406-3 (2024).

[9] Yu, Q., Liu, Y., Liu, X. & Yao. D and Yu. Y Experimental study on seepage flow patterns in heterogeneous low-permeability reservoirs. *J. Petroleum Explor. Prod. Technol.* **8**, 589–596. 10.1007/s13202-017-0354-y (2017).

[10] Khan, M. Y. & Mandal, A. The impact of permeability heterogeneity on water-alternating-gas displacement in highly stratified heterogeneous reservoirs. *J. Petroleum Explor. Prod. Technol.* **12**, 871–897. 10.1007/s13202-021-01347-3 (2022).

[11] Cao, C. et al. Non-Darcy seepage models of broken rock mass under changed hydraulic and porous structure. *Water* **17**, 1676. 10.3390/w17111676 (2025).

[12] Elnaggar, O. & Temraz, M. Lower paleozoic reservoir zonation into different flow units using turbulence factor and their relations to diagenises. *J. Petroleum Explor. Prod. Technol. V*. **8**, 351–361 (2018).

[13] Tiab, D. & Donaldson, E. C. : Petrophysics: Theory and Practice of Measuring Reservoir Rock and Fluid Transport Properties. 4th edition, Gulf Professional Publishing. (2015).

[14] Doveton, J. H. *Principles of Mathematical Petrophysics* (Oxford University Press, 2014). 10.1093/oso/9780199978045.001.0001

[15] Amaefule, J. O., Altunbay, M., Tiab, D., Kersey, D. G. & Keelan, D. K. : Enhanced reservoir description: Using core and log data to identify hydraulic (flow) units. SPE Annual Technical Conference and Exhibition, SPE-26436. (1993).

[16] Nabawy, B. S. *New Approaches in Reservoir Characterization Utilizing Conventional and Special Core Analyses: a Comprehensive Review* (Journal of Umm Al-Qura University for Applied Sciences, 2025).

[17] Nabawy, B. S., Rashed, M. A., Mansour, A. S. & Afify, W. S. M. Petrophysical and microfacies analysis as a tool for reservoir rock typing and modeling: rudeis Formation, off-shore October oil Field, Sinai. *Mar. Petroleum Geol.* **97**, 260–276 (2018a).

[18] Nabawy, B. S., Elgendy, N. T. H. & Gazia, M. T. Mineralogic and diagenetic controls on reservoir quality of paleozoic sandstones, Gebel El-Zeit, North Eastern Desert, Egypt. *Nat. Resour. Res.* **29**, 1215–1238 (2020).

[19] Elgendy, N. T. H., Abuamarah, B. A., Nabawy, B. S., Ghrefat, H. & Kassem, O. M. K. Pore fabric anisotropy of the Cambrian–Ordovician Nubia sandstone in the onshore Gulf of Suez, egypt: a surface outcrop analog. *Nat. Resour. Res.* **29**, 1307–1328 (2020).

[20] Abd, E., Aziz, E. A. & Gomaa, M. M. *Petrophysical and Seismic Evaluation of pre-rift Sediments of the Southern Gulf of Suez basin, Egypt* (Carbonates and Evaporites, 2024).

[21] Fathy, D., Lee, E. Y., Xiang, X., Fathi, E. & Sami, M. Petrophysical properties of the middle miocene sediments on the central Gulf of Suez, Egypt. *Front. Earth Sci.* **13**, 1592041. 10.3389/feart.2025.1592041 (2025).

[22] Poupon, A. & Leveaux, J. Evaluation of water saturation in Shaly formations. *Soc. Petrol. Eng. J.* **11** (06), 918–928. 10.2118/3360-PA (1971).

[23] Dykstra, H. & Parsons, R. L. *The Prediction of Oil Recovery by Water Flooding. Secondary Recovery of Oil in the United States* second edn, pp. 160–174 (API, 1950).

[24] El Sharawy, M. S. & Nabawy, B. S. Determining the porosity exponent m and lithology factor a for sandstones and their control by overburden pressure: a case study from the Gulf of Suez, Egypt. *AAPG Bull.* **102** (9), 1893–1910 (2018).

[25] Siddiqui, S., Okasha, M. T. M., Funk, J. J. & Al-Harbi, A. M. : New representative sample selection criteria for special core analysis. In Proceedings of the International Symposium of the Society of Core Analysts, Pau, France, 21–24; p. 11. (2003).

[26] Huang, H. & Ayoub, J. : Applicability of the Forchheimer equation for non-Darcy flow in porous media. *SPE J.*, 112–122. (2008).

[27] Barree, R. D. & Conway, M. W. *Beyond Beta Factors: a Complete Model for Darcy, Forchheimer, and trans-Forchheimer Flow in Porous Media* (SPE ATCE, 2004). SPE paper 89325.

[28] Smith, M. B. et al. : An investigation of non-Darcy flow effects on hydraulic fractured oil and gas well performance. SPE paper 90864. SPE ATCE, Houston. (2004).

[29] El Sharawy, M. S. Analysis of vertical heterogeneity measures based on routine core data of sandstone reservoirs. *Geosciences* **15**, 98. 10.3390/geosciences15030098 (2025).

[30] Elsanoose, A. et al. Characterization of a Non-Darcy flow and development of new correlation of NON-Darcy coefficient. *Energies* **15**, 7616. 10.3390/en15207616 (2022).

[31] Yoon, H. C. & Mallikarjunaiah, S. M. A steady Darcy–Brinkman–Forchheimer flow model in porous media: comparison study of non-Darcy flow models with viscous and inertial resistances and Forchheimer coefficient. *Eur. J. Mech. / B Fluids*. **114**, 204303 (2025).

[32] Katz, D. L. et al. *Handbook of Natural Gas Engineering* (McGraw-Hill Book Co., Inc., 1959).

[33] Geertsma, J. Estimating the coefficient of inertial resistance in fluid flow through porous media. *Soc. Petrol. Eng. J.* **14** (5), 445 (1974).

[34] Noman, R., Shrimanker, N. & Archer, J. S. : Estimation of the coefficient of inertial resistance in high-rate gas wells. SPE paper no. 14207. In: Presented at the 60th annual technical conference in Las Vegas, NV, 22–25. (1985).

[35] Aminian, K., Ameri, S. & Yussefabad, A. G. : A Simple and Reliable Method for Gas Well Deliverability Determination. SPE 111195, Kentucky, U.S.A. (2007).

[36] Schmalz, R. F. & Rahme, H. N. *The Variation of Waterflood Performance with Variation in Relative Permeability Characteristics* 223–244 (Drilling and Production Practice, American Petroleum Institute, 1950).

[37] Abeer, A., Abuhagaza, Marwa, Z. E., Sawy, B. S. & Nabawy. Integrated petrophysical and petrographical studies for characterization of reservoirs: a case study of Muglad Basin, North Sudan. *Environ. Earth Sci.* **80**, 171. 10.1007/s12665-021-09489-7 (2021).

[38] Winland, H. D. : Oil Accumulation in Response to Pore Size Changes. In: Field, W., Ed., Amoco Production Research Report, No. F72-G-25, Saskatchewan. (1972).

[39] Pittman, E. D. Relationship of porosity and permeability to various parameters derived from mercury injection-capillary pressure curves for sandstone. *AAPG Bull.* **76** (2), 191–198 (1992).

[40] Abuamarah, B. A. & Nabawy, B. S. : A proposed classification for the reservoir quality assessment of hydrocarbon-bearing sandstone and carbonate reservoirs: A correlative study based on different assessment petrophysical procedures. *J. Nat. Gas Sci. Eng.*, Volume 88. (2021).

[41] Maglio-Johnson, T. : Flow unit definition using petrophysics in a deep water turbidite deposit, Lewis Shale, Carbon County, Wyoming. Publishing M.Sc. thesis, Colorado School of Mines. (2000).

[42] El Sawy, M. Z., Abuhagaza, A. A., Nabawy, B. S. & Lashin, A. Rock typing and hydraulic flow units as a successful tool for reservoir characterization of Bentiu–Abu Gabra sequence, Muglad basin, Southwest Sudan. *J. Afr. Earth Sci.* **171**, 103961 (2020).

